# The comparative genomics of *Bifidobacterium callitrichos* reflects dietary carbohydrate utilization within the common marmoset gut

**DOI:** 10.1099/mgen.0.000183

**Published:** 2018-06-15

**Authors:** Korin Albert, Asha Rani, David A. Sela

**Affiliations:** ^1^​ Department of Food Science, University of Massachusetts, Amherst, MA, USA; ^2^​ Molecular and Cellular Biology Program, University of Massachusetts, Amherst, MA, USA; ^3^​ Department of Microbiology, University of Massachusetts, Amherst, MA, USA; ^4^​ Department of Microbiology and Physiological Systems, University of Massachusetts Medical School, Worcester, MA, USA

**Keywords:** bifidobacteria, comparative genomics, commensalism, gut microbiota, non-human primate

## Abstract

*Bifidobacterium* is a diverse genus of anaerobic, saccharolytic bacteria that colonize many animals, notably humans and other mammals. The presence of these bacteria in the gastrointestinal tract represents a potential coevolution between the gut microbiome and its mammalian host mediated by diet. To study the relationship between bifidobacterial gut symbionts and host nutrition, we analyzed the genome of two bifidobacteria strains isolated from the feces of a common marmoset (*Callithrix jacchus*), a primate species studied for its ability to subsist on host-indigestible carbohydrates. Whole genome sequencing identified these isolates as unique strains of *Bifidobacterium callitrichos*. All three strains, including these isolates and the previously described type strain, contain genes that may enable utilization of marmoset dietary substrates. These include genes predicted to contribute to galactose, arabinose, and trehalose metabolic pathways. In addition, significant genomic differences between strains suggest that bifidobacteria possess distinct roles in carbohydrate metabolism within the same host. Thus, bifidobacteria utilize dietary components specific to their host, both humans and non-human primates alike. Comparative genomics suggests conservation of possible coevolutionary relationships within the primate clade.

## Data Summary

1. Genome sequence data from isolates UMA51804 and UMA51805 are archived in the DDBJ/ENA/GenBank database (http://www.ncbi.nlm.nih.gov/genbank/) under accession numbers NWTW00000000 and NWTX00000000.

2. The genome sequence data from *B. callitrichos* JCM 17296^T^ used in this study are available in the GenBank database (http://www.ncbi.nlm.nih.gov/genbank/) under accession number GCA_000741175.1.

**Impact Statement**

Microbial commensals colonize mammalian gastrointestinal tracts to assemble into communities known as microbiomes. Bacterial commensals contribute to host physiological and homeostatic operations. It has been postulated that microbial communities coevolved with their hosts, with various degrees of scientific evidence in support of this. Accordingly, bifidobacteria colonize nursing infants early in life due in part to their ability to subsist on human milk oligosaccharides. Bifidobacteria are also members of adult gut microbiomes, albeit to a lesser extent. Herein we describe a comparative genomic analysis of three *Bifidobacterium callitrichos* strains isolated from common marmoset feces. This was performed to investigate the coevolution of gut commensals with their host mediated by dietary components. In this instance, the common marmoset subsists on oligosaccharide-rich tree gums, and thus the genomic analysis focused on carbohydrate metabolism. Moreover, we tested genomics-derived hypotheses by verifying carbohydrate-related phenotypes. This included evaluating the capacity to utilize milk oligosaccharides as a sole carbohydrate source. This is significant as this trait is shared by select bifidobacterial species that colonize human infants. Interestingly, all three *B. callitrichos* strains exhibited the milk oligosaccharide utilization phenotype. This investigation provides a foundation for studies of the evolutionary relationship between mother’s milk, the infant, and its gut microbiome.

## Introduction

The genus *Bifidobacterium* contains anaerobic, non-spore-forming, rod-shaped bacteria that present a bifurcated, termed ‘bifid’, morphology under certain growth conditions in some species [1]. Bifidobacteria are commonly found in animal gastrointestinal tracts including mammals, birds, and insects [2–5]. These bacteria have been extensively characterized regarding their ability to metabolize host-indigestible carbohydrates, which often enables their persistence within the gut environment [6–8]. Previous genomic analyses identified genomic signatures underlying oligosaccharide utilization by bifidobacteria [9–11]. It is noteworthy that bifidobacteria associated with different human stages of development have divergent metabolic capabilities. This includes the infant-colonizing *Bifidobacterium longum* subsp. *infantis* that metabolizes indigestible oligosaccharides secreted in human milk. This rare phenotype has been linked to a 40 kb gene cluster dedicated to this function [12–14].

The gut microbiome is postulated to have coevolved simultaneously with its host gastrointestinal environment modulated by intrinsic and extrinsic factors such as diet [15–17]. Thus, the study of extant primates provides an opportunity to analyze potential coevolutionary relationships between hosts and microbes. In recent years, there has been an increase in the isolation and characterization of novel bifidobacterial species from non-human primate feces. Since 2012, at least 17 novel species of bifidobacteria have been described from non-human primates, with five from the common marmoset (*Callithrix jacchus*) feces [18–27]. Marmosets provide an interesting model to study the evolution of diet with gut microbiota, as they are one of the few mammals that subsist on indigestible oligosaccharides found in tree gums or hardened saps [28–32]. This subsistence strategy is relatively rare among mammals, with a very small fraction of mammalian species able to consume plant gums to some extent (*n=*94), but common among primates (*n=*78) [33]. The common marmoset is one of 27 mammalian species considered to be obligate exudivores [33]. The exudatory diet of common marmosets provides a rich source of plant β-linked polysaccharides, consisting largely of galactose, arabinose, and rhamnose [34–37].

*Bifidobacterium callitrichos* JCM 17296^T^ is a facultative anaerobic gastrointestinal symbiont of the common marmoset first isolated in 2012 [19]. A phylogenetic analysis of the family *Bifidobacteriaceae* using 404 clusters of orthologous groups (COGs) of proteins inferred that *B. callitrichos* JCM 17296^T^ lies within a clade that includes human-associated *Bifidobacterium breve* LMG 13208, *Bifidobacterium angulatum* LMG 11039, *Bifidobacterium longum* subsp. *longum* LMG 13197, *Bifidobacterium longum* subsp. *infantis* ATCC 15697 and *Bifidobacterium longum* subsp. *suis* LMG 21814, and several non-human primate-associated species [22]. Furthermore, a comparative genomic survey of the genus *Bifidobacterium* noted that the genome of *B. callitrichos* JCM 17296^T^ is larger than the expected average and may employ metabolic pathways lost from other bifidobacteria. The present study provides a comparative genomic and phenotypic analysis of *B. callitrichos* JCM 17296^T^ and two *B. callitrichos* strains recently isolated from the feces of a captive common marmoset.

## Methods

### Identification of microbial community members within source fecal sample

Total DNA from the marmoset fecal sample was isolated using the MO BIO PowerSoil DNA Isolation Kit as per the manufacturer’s instructions (Qiagen). The sequencing library was prepared with the extracted DNA using the Illumina 16S rRNA Metagenomic Sequencing Library Preparation protocol. Sequencing was performed on an Illumina MiSeq device using the 600-cycle MiSeq V3 reagent kit. Reads underwent quality filtering and were analyzed using the QIIME 1 pipeline [38–42].

### Bacterial isolation from fecal specimens

A fecal sample was collected from a 6-year-old female marmoset housed at the University of Massachusetts Amherst in Amherst, MA, USA. The animals were cared for in accordance with the guidelines published in the *Guide for the Care and Use of Laboratory Animals*, 8th edition. The study was approved by the UMass Institutional Animal Care and Use Committee. The fresh fecal sample was mixed with 5 ml of sterile peptone water and spread onto bifidobacteria-specific medium (BSM) agar, which consists of de Mann Rogosa Sharpe (MRS) agar (Difco), 0.05 % (w/v) l-cysteine (Sigma Aldrich) and 0.05 % (w/v) mupirocin (AppliChem Panreac) [43]. Agar plates were incubated at 37 °C for 24 h in a Coy anaerobic chamber maintained with a gas mix of 7 % H_2_, 10 % CO_2_, and N_2_ to balance (Coy Laboratory Products). Individual colonies were selected and grown in BSM broth for 12 h under the same conditions. Liquid cultures were preserved as freezer stocks at −80 °C in a 25 % (v/v) glycerol solution. Isolates were initially screened via colony PCR with bifidobacteria-specific primers (Bif164f: GGGTGGTAATGCCGGATG, Bif662r: CCACCGTTACACCGGGAA) amplifying a 550 bp fragment of the 16S rRNA [44–46]. PCR products were purified using the QIAquick PCR Purification Kit (Qiagen) and sequenced by Genewiz using Sanger DNA Sequencing (Genewiz). The resulting sequences were used to infer phylogenetic relationships in mega7 using the EzBioCloud 16S rRNA gene sequence database to provide an initial screen [47, 48]. Bifidobacterial isolates were further confirmed using the fructose 6-phosphate phosphoketolase assay. This colorimetric assay determines the presence of a unique phosphoketolase involved in the bifidobacterial-specific fermentative pathway, or ‘bifid shunt’ [49–51].

### Whole genome sequencing of bacterial isolates

Genomic DNA was extracted using the MasterPure Gram Positive DNA Purification Kit [Epicentre (an Illumina Company)] and then further processed using the Genomic DNA Clean and Concentrator (Zymo Research) according to the manufacturer’s instructions. DNA quality and quantity were determined using a NanoDrop 2000 Spectrophotometer and a Qubit 2.0 Fluorometer (Thermo Fisher Scientific), respectively. Sequencing libraries were prepared with the Nextera XT 150 bp paired-end library preparation kit according to the manufacturer’s instructions (Illumina). Subsequently, whole genome sequencing was performed on an Illumina NextSeq using the NextSeq V2 reagent kit. Reads were assembled *de novo* using the SPAdes Genome Assembler 3.9.1 with default stringency parameters [52]. Assemblies were performed on the Massachusetts Green High Performance Computing Cluster (www.mghpcc.org).

### Bacterial phylogeny inference

Following whole genome sequencing, phylogenetic analysis was performed using bcgTree, which infers a maximum-likelihood phylogeny using the concatenated sequences of 107 single-copy core genes with 1000 bootstraps [53]. The hmmer v3.1b2 hmmsearch tool was used to locate the single-copy genes amino acid sequences in each genome, muscle 3.8.31 to create a multiple sequence alignment based on the resulting presence/absence table, Gblocks 0.91b to refine the alignment, and RaxML 8.2.4 to build the maximum-likelihood phylogenetic tree [53–58]. Reference *Bifidobacterium* genomes were obtained from the NCBI RefSeq database under the following accession numbers: NZ_MWWV00000000.1 (*B. tissieri* JCM 30798^T^), NZ_AZMV00000000.1 (*B. moukalabense* JCM 18751^T^), NZ_MWWY00000000.1 (*B. hapali* JCM 30799^T^), NZ_JDUU00000000.1 (*B. biavatii* JCM 17299^T^), NZ_BDIS00000000.1 (*B. lemurum* JCM 30168^T^), NZ_MWWZ00000000.1 (*B. eulemuris* JCM 30801^T^), NZ_MWWW00000000.1 (*B. myosotis* JCM 30796^T^), NZ_JGZK00000000.1 (*B. reuteri* JCM 17295^T^), NZ_JGZN00000000.1 (*B. saguini* JCM 17297^T^), NZ_BCFK00000000 (*B. aesculapii* JCM 18761^T^), NZ_JGZP00000000 (*B. stellenboschense* JCM 17298^T^), and NZ_JGYS00000000 (*B. callitrichos* JCM 17296^T^). [59]. Final phylogenetic trees were visualized and formatted using FigTree v1.4.3 and Phylo.io [60, 61].

### Sequencing data analysis

Initial gene model predictions and annotations were performed using the Rapid Annotation using Subsystem Technology (RAST) annotation server [62–64]. Genes were sorted into functional categories using the SEED database through the RAST annotation server, and the percentage of genes belonging to each functional category was calculated relative to the total number of genes in each genome. The genomes of isolates UMA51804 and UMA51805 were submitted for auto-annotation with the Department of Energy’s Joint Genome Institute (JGI) Integrated Microbial Genomes (IMG) Microbial Genome and Metagenome Expert Review Data Submission platform [65]. RAST protein-coding gene predictions were assigned Kyoto Encyclopedia of Genes and Genomes (KEGG) orthology identifications using BlastKOALA [66]. KEGG Orthology (KO) identifiers were converted to Enzyme Commission (EC) numbers using the Carbohydrate Metabolism KEGG reference pathways [67–69]. In addition, genes associated with carbohydrate metabolism were identified using the Carbohydrate-Active Enzymes (CAZy) database (http://www.cazy.org/) via the Database for Automated Carbohydrate-Active Enzyme Annotation (dbCAN) web tool [70, 71]. The presence and absence of genes within each genome was determined using Venny 2.1.0. [72]. Amino acid sequence analysis to determine potential extracellular protein localization was performed with PSORTb version 3.0.2 [73].

### Metabolic phenotyping of fermentative substrates

Bacterial strains were tested for their ability to grow on specific carbohydrates as a sole carbon source. Briefly, a 1 % (v/v) overnight culture of each isolate, including biological and technical triplicates, was grown on modified MRS (mMRS) media containing 2 % (w/v) of each carbon substrate: arabinose, cellobiose, fructose, galactose, glucose, acacia gum, arabinoxylan, cranberry xyloglucans (provided by Ocean Spray), lactose, maltose, mannose, *N*-acetylglucosamine (NAG), rhamnose, sorbitol, tamarind gum, trehalose, xylan and xylose. Biomass production was estimated by measuring the optical density at 600 nm (OD_600_). To determine final OD_600_, mMRS was inoculated with an overnight culture at a concentration of 1 % and then incubated for 72 h at 37 °C under anaerobic conditions. Negative controls consisted of inoculated media in the absence of a carbon substrate. Two-way analysis of variance was performed using GraphPad Prism version 6 (GraphPad Software, www.graphpad.com). In addition, the ability of each isolate to grow on 2′-fucosyllactose and pooled human milk oligosaccharides (HMOs) was assayed using a PowerWave HT Microplate Spectrophotometer (BioTek). Overnight cultures grown in MRS broth were used to inoculate mMRS at a concentration of 1 %. Isolates then grew anaerobically for 40 h at 37 °C with shaking and OD_600_ measurements were taken every 15 min. OD_600_ values were plotted over time to create a logarithmic growth curve using GraphPad Prism.

## Results

### Microbial diversity in the marmoset gut is dominated by *Proteobacteria* and *Firmicutes*

**Fig. 1.**
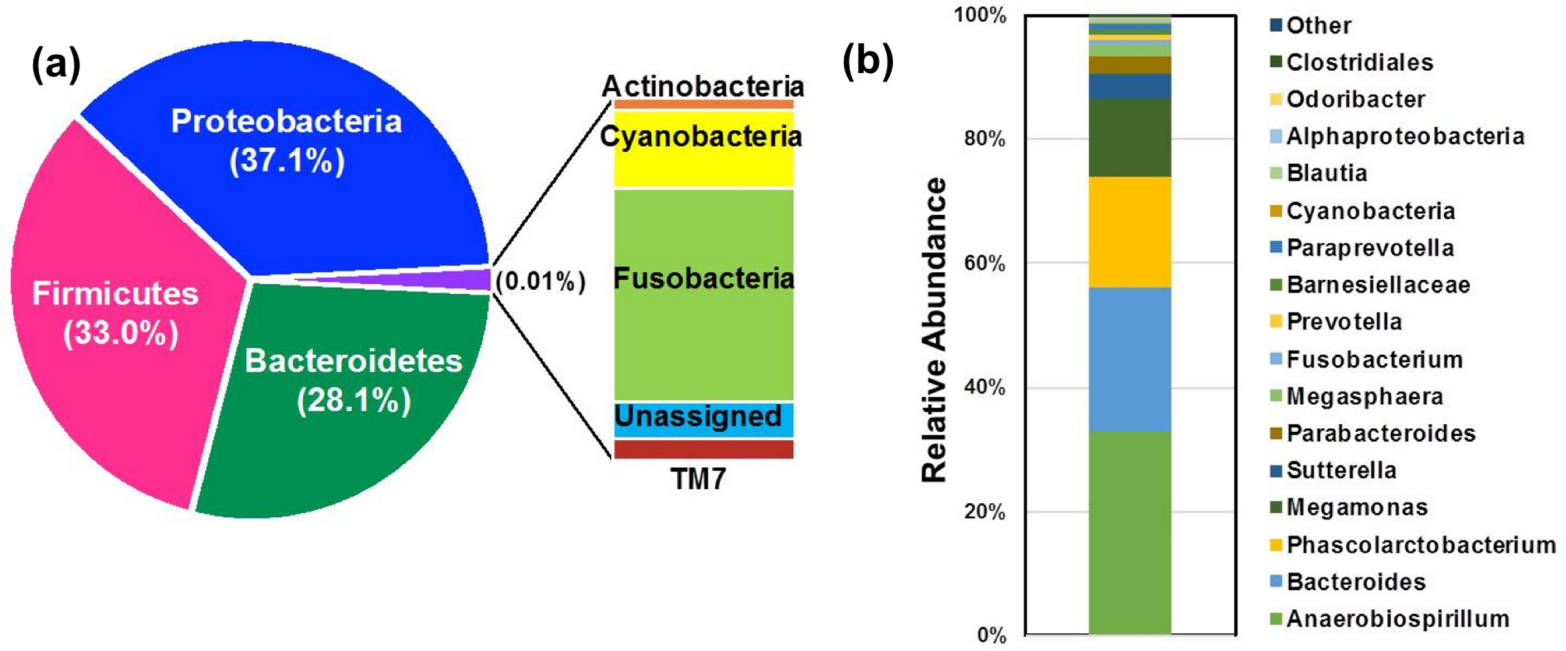
Relative taxon abundance in the marmoset gut. Shown are pie and bar chart representations of the relative abundance values at the (a) phylum and (b) genus level of microbial diversity in the marmoset gut using 16S rRNA gene sequencing. Each color represents a phylum (a) and the top 16 genera (b) identified (at >0.2 % abundance) in the marmoset gut.

The microbial community structure in the marmoset gut was determined from the source fecal sample using 16S rRNA amplicon sequencing. This sample was dominated by three bacterial phyla: *Proteobacteria* (37.1 %), *Firmicutes* (33.0 %), and *Bacteroidetes* (28.1 %). Other phyla at low abundance (<0.01 %) were members of *Actinobacteria*, *Cyanobacteria*, *Fusobacteria*, *TM7,* and unassigned classes of bacteria (Fig. 1a). A detailed analysis at the operational taxonomic unit (OTU) level identified the top 16 abundant bacterial genera in this sample, followed by other less abundant (<0.2 %) genera (Fig. 1b). The most abundant genera identified were *Anaerobiospirillum* (32.5 %), *Bacteroides* (22.8 %), *Phascolarctobacterium* (17.8 %) and *Megamonas* (12.4 %). The genus *Anaerobiospirillum* consists of Gram-negative, anaerobic, spiral-shaped rods which are known to be the part of the normal gastrointestinal microbiota of dogs and cats [74, 75]. *Bacteroides* species are important members of the human and animal gut microbiota identified from feces (representing approximately 30 % of the cultured species) [76, 77]. *Phascolarctobacterium* are Gram-negative, non-spore-forming, saccharolytic *Firmicutes*, which could produce short-chain fatty acids and have been identified in human and koala feces [78]. Members of the genus *Megamonas* have been associated with obesity and glucose tolerance in the human gut microbiome, but their role in the marmoset gut is not clearly understood [79, 80].

Interestingly, the genus *Bifidobacterium* was detected at low abundance (0.04 %) in the feces of this particular marmoset adult. However, low abundance does not necessarily reflect the functional importance of this genus within the marmoset microbiome. Based on the increased carbohydrate metabolism of bifidobacteria and the high carbohydrate diet of the common marmoset, we chose to isolate and analyze bifidobacterial strains present.

### General genome characteristics

**Fig. 2.**
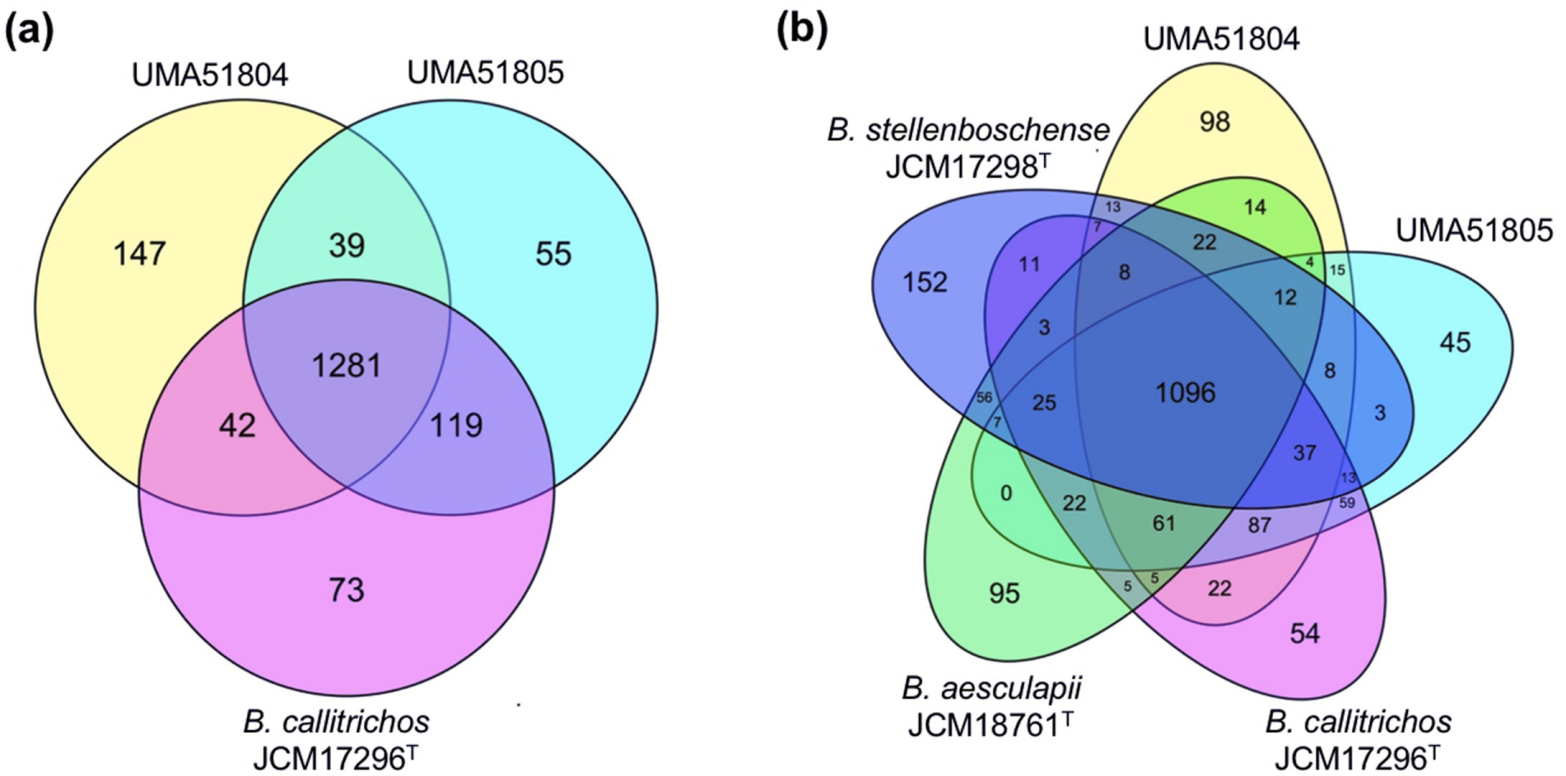
Genomic diversity of *B. callitrichos*. Venn diagrams showing the number of genes shared and unique between (a) *B. callitrichos* JCM 17296^T^, UMA51804, and UMA51805, and (b) genes shared between *B. callitrichos* strains (UMA51804, UMA51805) and closely related species *B. aesculapii* JCM 18761^T^ and *B. stellenboschense* JCM 17298^T^. The *B. callitrichos* strains UMA51804 and UMA51805 shared a higher number of genes with *B. callitrichos* JCM 17296^T^ than with each other and other type strains included in the analysis.

The median genome size of *B. callitrichos* JCM 17296^T^ is 2.88 Mb with a G+C content of 63.6 mol% and 2230 protein-coding genes in 33 scaffolds [19, 22, 81, 82]. Isolates UMA51804 and UMA51805 have genome sizes of 3.04 and 2.78 Mb with G+C contents of 64.5 and 63.6 mol%, respectively (Table 1). Sequence similarity between UMA51804 and UMA51805 is depicted visually via a dot plot in Fig. S1 (available in the online version of this article). Interestingly, UMA51804 encodes a higher number of protein-coding genes (2528) than UMA51805 (2228) and *B. callitrichos* JCM 17296^T^ (2230), which is consistent with its larger genome size. The RAST genome annotation for UMA51804 also contains a larger percentage of hypothetical genes (31.59 %) compared with UMA51805 (23.62 %) and JCM 17296^T^ (26.25 %). Genes unique to UMA51804 are not dominated by any particular functional category and include CRISPR- and phage-associated proteins. Average genomic nucleotide identity between UMA51804 and JCM 17296^T^ was 91.41 %, which suggests that UMA51804 may not belong to the same species *sensu stricto*. An alternative explanation is that this isolate may represent a different subspecies from UMA51805 and *B. callitrichos* JCM 17296^T^ [83]. Utilization of the alignment fraction method for inter-species determination produced a 40 % probability that UMA51804 represents a separate species from *B. callitrichos* [84]. UMA51804 and UMA51805 share 39 (2.2 %) genes not found in JCM 17296^T^, and the core genome of all three strains consists of 1281 genes (Fig. 2a). The closely related strains *Bifidobacterium aesculapii* JCM 18761T and *Bifidobacterium stellenboschense* JCM 17298^T^ have 95 and 152 genes, respectively, not identified in the analyzed *B. callitrichos* genomes by the RAST SEED database (Fig. 2b).

**Table 1.** General genome characteristics of *B. callitrichos* strains and closely related species

Characteristic	*B. callitrichos* UMA51804	*B. callitrichos* UMA51805	*B. callitrichos* JCM 17296^T^	*B. aesculapii* JCM 18761^T^	*B. stellenboschense* JCM 17298^T^	*B. angulatum* JCM 1252^T^
Isolation source	*Callithrix jacchus* feces	*Callithrix jacchus* feces	*Callithrix jacchus* feces	*Callithrix jacchus* feces	*Saguinus midas* feces	Adult human feces
Status	Draft	Draft	Draft	Draft	Draft	Complete
Number of scaffolds	92	88	40	93	40	1
Median genome size (Mb)	3.02	2.76	2.87	2.69	2.81	2.01
Median G+C content (mol%)	64.5	63.6	63.5	64.8	65.3	59.4
Median protein count	2465	2200	2230	1989	2102	1520
GenBank assembly accession no.	GCA_003024955.1	GCA_003024945.1	GCA_000741175.1	GCA_001417815.1	GCA_000741785.1	GCA_001025155.1

### Phylogenetic relatedness within the genus *Bifidobacterium*

**Fig. 3.**
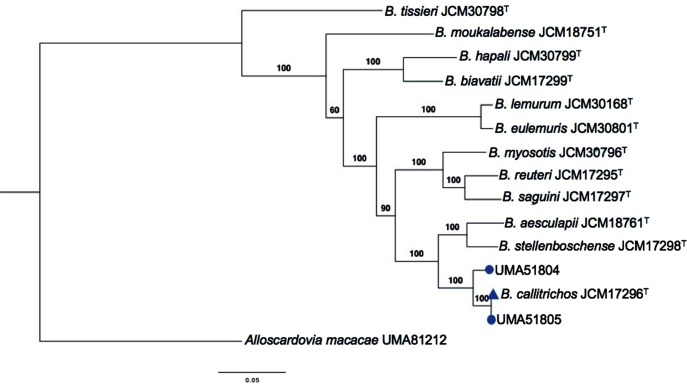
Maximum-likelihood phylogenetic tree of members of the genus *Bifidobacterium* originating from non-human primates. The tree was created using the bcgTree pipeline with 107 essential single-copy core genes, found in a majority of bacteria, using hidden Markov models based on a partitioned maximum-likelihood analysis. Bootstrap confidence values were obtained with 1000 resamplings and are provided at branch points. The scale bar represents the number of amino acid substitutions per site. Microbial isolates identified in this study are shown as blue circles closely clustered with *B. callitrichos* JCM 17296^T^ (blue triangle). *Alloscardovia macacae* (UMA81212) was used as the outgroup species within the family *Bifidobacterium.*

Initial identification of the isolates as members of *B. callitrichos* used 16S rRNA gene sequences to create a phylogenetic model (Fig. S2). The phylogenetic tree placed UMA51804 and UMA51805 as closely related to *B. callitrichos* JCM 17296^T^. The phylogenetic relationships inferred from the whole genome sequences of *B. callitrichos* JCM 17296^T^, UMA51804, UMA51805, and other bifidobacteria hosted within non-human primates are depicted in Fig. 3. UMA51804 clusters separately from UMA51805 and *B. callitrichos* JCM 17296^T^ within an individual clade supported by a bootstrap value of 100, which supports the initial 16S rRNA phylogenetic inference (Fig. S2). The UMA51804, UMA51805, and JCM 17296^T^ clade shares a node with *B. aesculapii* JCM 18761^T^ and *B. stellenboschense* JCM 17298^T^, which were isolated from feces of an infant common marmoset and a red-handed tamarin, respectively [19, 21]. *B aesculapii* JCM 18761^T^ and *B. stellenboschense* JCM 17298^T^, along with many other non-human primate bifidobacteria, have not been studied extensively. Overall, additional strains from these primates will provide greater phylogenetic resolution of evolutionary relatedness between isolates from the same host.

### The core genome of *B. callitrichos* contains carbohydrate utilization genes associated with host diet

Table 2 lists the names and corresponding EC numbers of predicted genes found in the top three highest represented KEGG carbohydrate metabolic pathways. All three *B. callitrichos* strains exhibit genes coding for ATP-binding cassette (ABC)-type transporters predicted to import exogenous carbohydrate molecules. Specifically, the core genome contains transporters of the multiple sugar metabolism system. These are ABC transporters including ATP-binding proteins, permeases, and substrate binding proteins (see Table S1 for locus tags). This fits with previous studies of bifidobacteria demonstrating the importance of ABC transporters for the import of exogenous carbohydrates into the cell [85, 86]. In addition, several phospho-transferase systems (PTSs) are represented within the genomes. For instance, the core genome contains subunits IIA, IIB, and IIC of the PTS transporter system for the import of lactose and cellobiose, and also has the components of the PTSs specific to trehalose, NAG, and β-glucoside (see Table S2 for locus tags). Bifidobacterial carbohydrate metabolism could involve the secretion of extracellular enzymes which bind or alter carbohydrate structures prior to uptake [87, 88]. Using the CAZy database and the PSORTb subcellular localization tool, three major categories of carbohydrate-active enzymes, glycosyl hydrolase families 25 and 51, carbohydrate esterase family 4, and carbohydrate-binding molecule family 22, were predicted as extracellularly targeted in all three genomes (Tables S3 and S4). The representation of all carbohydrate-active enzyme families is similar among the three strains (Fig. S3 and Table S3).

**Table 2.** KEGG carbohydrate metabolic pathway genes shared by *B. callitrichos* JCM 17296^T^ and isolates UMA51804 and UMA51805 identified in this study. Genes shown are those for which a locus tag in each genome could be identified. Pathways are highlighted based on the different metabolic categories.

**KEGG carbohydrate metabolic pathway**	**Gene name**	**EC**	**UMA51804 locus tag**	**UMA51805 locus tag**	**JCM 17296^T^ locus tag**
**Galactose metabolism**	UTP—glucose 1-phosphate uridylyltransferase	2.7.7.9	COO72_RS10475	CPA40_RS09980	BCAL_RS08755
	Aldose 1-epimerase	5.1.3.3	COO72_RS03125	CPA40_RS00475	BCAL_RS03445
	Galactokinase	2.7.1.6	COO72_RS01650	CPA40_RS06710	BCAL_RS05755
	UDP-glucose 4-epimerase	5.1.3.2	COO72_RS04485	CPA40_RS02855	BCAL_RS02635
	l-Ribulose-5-phosphate 4-epimerase	5.1.3.4	COO72_RS03920	CPA40_RS04240	BCAL_RS05325
	*N*-Acylglucosamine 2-epimerase	5.1.3.8	COO72_RS09925	CPA40_RS00610	BCAL_RS02285
**Starch and sucrose metabolism**	α,α-Phosphotrehalase	3.2.1.93	COO72_RS08005	CPA40_RS06370	BCAL_RS06195
	Protein-Nπ-phosphohistidine—trehalose phosphotransferase	2.7.1.201	COO72_RS08010	CPA40_RS06365	BCAL_RS06200
	β-Fructofuranosidase	3.2.1.26	COO72_RS02000	CPA40_RS06035	BCAL_RS10725
	α-Glucosidase	3.2.1.20	COO72_RS09510	CPA40_RS01270	BCAL_RS0830
	Amylosucrase	2.4.1.4	COO72_RS05900	CPA40_RS04855	BCAL_RS04290
	Sucrose phosphorylase	2.4.1.7	COO72_RS10000	CPA40_RS07310	BCAL_RS06670
	UTP—glucose-1-phosphate uridylyltransferase	2.7.7.9	COO72_RS10475	CPA40_RS09980	BCAL_RS08755
	α-Amylase	3.2.1.1	COO72_RS11340	CPA40_RS02640	BCAL_RS02400
	β-Glucosidase	3.2.1.21	COO72_RS09740	CPA40_RS01270	BCAL_RS01445
	Isoamylase	3.2.1.68	COO72_RS04240	CPA40_RS05215	BCAL_RS04710
	Glucose-1-phosphate adenylyltransferase	2.7.7.27	COO72_RS01125	CPA40_RS05455	BCAL_RS09705
	1,4-α-Glucan branching enzyme	2.4.1.18	COO72_RS03710	CPA40_RS0112	BCAL_RS04065
	4-α-Glucanotransferase	2.4.1.25	COO72_RS08655	CPA40_RS05200	BCAL_RS04695
	Glycogen phosphorylase	2.4.1.1	COO72_RS08360	CPA40_RS09325	BCAL_RS09090
	Starch synthase (maltosyl-transferring)	2.4.99.16	COO72_RS06885	CPA40_RS03955	BCAL_RS11605
	Phosphoglucomutase (α-d-glucose-1,6-bisphosphate-dependent)	5.4.2.2	COO72_RS10095	CPA40_RS03335	BCAL_RS04990
	Protein-Nπ-phosphohistidine—cellobiose phosphotransferase	2.7.1.205	COO72_RS05245	CPA40_RS08235	BCAL_RS07995
**Amino sugar and nucleotide sugar metabolism**	UDP-*N*-acetylmuramate dehydrogenase	1.3.1.98	COO72_RS09690	CPA40_RS03405	BCAL_RS04925
	UDP-*N*-acetylglucosamine 1-carboxyvinyltransferase	2.5.1.7	COO72_RS11030	CPA40_RS10465	BCAL_RS06425
	UDP-*N*-acetylglucosamine diphosphorylase	2.7.7.23	COO72_RS04345	CPA40_RS08380	BCAL_RS00645
	*N*-Acetylglucosamine-6-phosphate deacetylase	3.5.1.25	COO72_RS10300	CPA40_RS06275	BCAL_RS02715
	Glucosamine-1-phosphate *N*-acetyltransferase	2.3.1.157	COO72_RS04345	CPA40_RS08380	BCAL_RS00645
	Phosphoglucosamine mutase	5.4.2.10	COO72_RS05530	CPA40_RS04580	BCAL_RS02085
	Glucosamine-6-phosphate deaminase	3.5.99.6	COO72_RS08105	CPA40_RS05730	BCAL_RS02710
	Glutamine—fructose-6-phosphate transaminase (isomerizing)	2.6.1.16	COO72_RS05020	CPA40_RS04675	BCAL_RS02180
	Non-reducing end α-l-arabinofuranosidase	3.2.1.55	COO72_RS05470	CPA40_RS04135	BCAL_RS02145
	Glucose-6-phosphate isomerase	5.3.1.9	COO72_RS12210	CPA40_RS08670	BCAL_RS05265
	UDP-glucose 6-dehydrogenase	1.1.1.22	COO72_RS07800	CPA40_RS09840	BCAL_RS07900
	Phosphoglucomutase (α-d-glucose-1,6-bisphosphate-dependent)	5.4.2.2	COO72_RS10095	CPA40_RS03335	BCAL_RS04990
	UTP—glucose-1-phosphate uridylyltransferase	2.7.7.9	COO72_RS10475	CPA40_RS09980	BCAL_RS08755
	UDP-glucose 4-epimerase	5.1.3.2	COO72_RS04485	CPA40_RS02855	BCAL_RS02635
	Galactokinase	2.7.1.6	COO72_RS01650	CPA40_RS06710	BCAL_RS05755
	UDP-glucose—hexose-1-phosphate uridylyltransferase	2.7.7.12	COO72_RS11900	CPA40_RS05725	BCAL_RS05750
	UDP-galactopyranose mutase	5.4.99.9	COO72_RS07765	CPA40_RS11110	BCAL_RS07940
	Phosphoglucomutase (α-d-glucose-1,6-bisphosphate-dependent)	5.4.2.2	COO72_RS10095	CPA40_RS03335	BCAL_RS04990
	Glucose-1-phosphate adenylyltransferase	2.7.7.27	COO72_RS01125	CPA40_RS05455	BCAL_RS09705

The *B. callitrichos* core genome is predicted to encode several genes in diverse carbohydrate metabolic pathways. Genomic locus tags are defined with the following convention: the UMA51804 locus tag prefix is COO72_, the UMA51805 locus tag prefix is CPA40_, and the JCM 17296^T^ locus tag prefix is BCAL_. All three genomes contain predicted l-arabinose isomerase genes (COO72_RS01355, CPA40_RS10585, BCAL_RS05330), which could convert arabinose to ribulose and galactose to tagatose. The presence of genes associated with galactose and arabinose metabolism is consistent with the major carbon constituents of tree gums found in common marmoset habitats [34]. α,α-phosphotrehalase (COO72_RS08005, CPA40_RS06370, BCAL_RS06195), which converts trehalose 6-phosphate to d-glucose 6-phosphate, may reflect the marmoset's utilization of alternative nutritive sources in addition to gums. This includes insects that contain trehalose in their hemolymph [89]. Furthermore, several dietary carbohydrates are predicted to be utilized by *B. callitrichos* through hydrolysis or interconversions to d-glucose including maltose and sucrose via α-glucosidase (COO72_RS09510, COO72_RS09740; CPA40_RS00310, CPA40_RS00625; BCAL_RS03280, BCAL_RS03615), cellobiose via β-glucosidase (COO72_RS03545, COO72_RS09740, COO72_RS05215; CPA40_RS01270, CPA40_RS06850, CPA40_RS08995, CPA40_RS02570; BCAL_RS04215, BCAL_RS10930, BCAL_RS01445), melibiose via α-galactosidase (COO72_RS10210, COO72_RS10225, COO72_RS00655, COO72_RS12035; CPA40_RS10210, CPA40_RS10230, CPA40_RS10525; BCAL_RS06730, BCAL_RS08175, BCAL_RS08225), and lactose via β-galactosidase (COO72_RS01410, COO72_RS05460, COO72_RS07355, COO72_RS08450, COO72_RS08505; CPA40_RS04650, CPA40_RS05890, CPA40_RS09155, CPA40_RS09460 CPA40_RS09665, CPA40_RS09775, CPA40_RS02920; BCAL_RS02155, BCAL_RS02705, BCAL_RS05420, BCAL_RS07405, BCAL_RS10570).

Recently, a genomic analysis of *B. breve* revealed ~15 kb mannitol/sorbitol utilization cluster in several strains [90]. The genome of *B. callitrichos* JCM 17296^T^ has a similar cluster of ~12 kb which contains sorbitol dehydrogenase (BCAL_RS04735), alcohol dehydrogenase (BCAL_RS04755), lactoylglutathione lyase (BCAL_RS00215), an *araC* family transcriptional regulator (BCAL_RS04730), an ROK family transcriptional regulator (BCAL_RS04750), and an MFS superfamily sugar alcohol transporter (BCAL_RS04740). All of these components are clustered in UMA51804 with minor divergence to adjacent genes, and on two separate contigs in UMA51805. In addition, all three genomes contain α-galactosidases which could also catalyse the metabolism of galactinol, d-*myo*-inositol, sorbitol, melibitol, and glycerol.

### Variation in gene representation between isolates may contribute to carbohydrate metabolic diversity

**Fig. 4.**
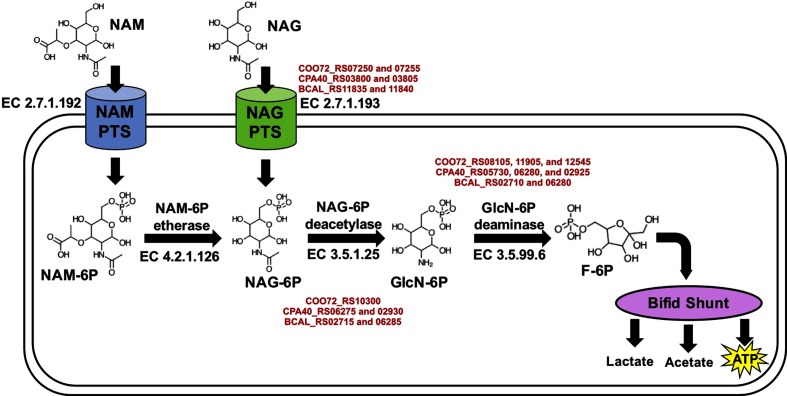
Proposed mechanism for the utilization of *N*-acetylmuramic acid (NAM) and *N*-acetylglucosamine (NAG). NAM and NAG serve as the precursors for fructose 6-phosphate, which feeds into the bifid shunt pathway. The proposed mechanism is depicted to show the structure and EC number for the enzymes involved in the pathway. Text in red represents the locus tags for UMA51804, UMA51805, and JCM 17296^T^, in that order.

*N*-Acetylglucosamine (i.e. NAG or GlcNAc) is a moiety incorporated into peptidoglycan, which is a major constituent of the bacterial cell wall [91]. In addition, NAG is incorporated within milk oligosaccharides, which could be utilized as a fermentative substrate by *B. longum* subsp. *infantis* and other bifidobacterial species [92]. Extracellular NAG, regardless of source, could be transported into the cell by NAG PTS transporter systems and enter the bifid shunt after several modifications. All three genomes include a NAG-specific PTS intracellular transporter system (Fig. 4, Table S2). Interestingly, a KEGG pathway analysis identified a PTS transporter for a related molecule, *N*-acetylmuramic acid (NAM), in UMA51804 and UMA51805. NAM has the same structure as NAG but with an ester group bound to the oxygen at the C3 position. *B. callitrichos* metabolism of NAM is unclear, but it is possible that NAM is further catabolized to fructose 6-phosphate to enter the bifid shunt. NAM 6-phosphate (-6P) and NAG-6P are typically interconverted in the cytoplasm via NAM-6P etherase (EC 4.2.1.126), although this gene sequence was not detected in any of the *B. callitrichos* genomes. It is possible that NAM-6P is converted to NAG-6P via an as yet uncharacterized mechanism.

The *B. callitrichos* UMA51804 genome is significantly larger than that of UMA51805 and JCM 17296^T^ as it contains more protein-coding loci. This has not translated to an increased percentage of carbohydrate-related genes in UMA51804 (Fig. S4). However, UMA51804 does have unique carbohydrate-related genes, for example subunits IIA (COO72_RS02120), IIB (COO72_RS02125), and IIC (COO72_RS02130) of the fructose-specific PTS. In addition, UMA51804 has several genes predicted to be specific to HMO utilization. Of note, the presence of lacto-N-biose phosphorylase (COO72_RS10625), a key enzyme in the lacto-N-biose pathway, suggests that UMA51804 may have the ability to metabolize marmoset milk oligosaccharides [12, 93, 94]. This enzyme phosphorylates the disaccharides lacto-N-biose and galacto-N-biose, carbohydrate residues that comprise milk and mucin glycans, which permits further hydrolysis and catabolism of the substrate [95, 96]. The UMA51804 genome encodes predicted galacto-N-biose/lacto-N-biose ABC transporter components (substrate-binding protein: COO72_RS10640, permeases: COO72_RS10635 and COO72_RS10630) and l-fuconolactone hydrolase (COO72_RS01745), which are active on milk glycans, suggesting a utility early in development of the marmoset. UMA51804 also possesses a predicted *N*-acetylneuraminate lyase (COO72_RS01755), which could catalyze hydrolysis of negatively charged milk oligosaccharides (i.e. sialylated glycans) [97, 98]. The potential for UMA51804 to utilize marmoset milk oligosaccharides provides a compelling hypothesis related to the role of UMA51804 within the developing marmoset gut and later life stages.

### *B. callitrichos* strains ferment several dietary carbohydrates available to the marmoset

**Fig. 5.**
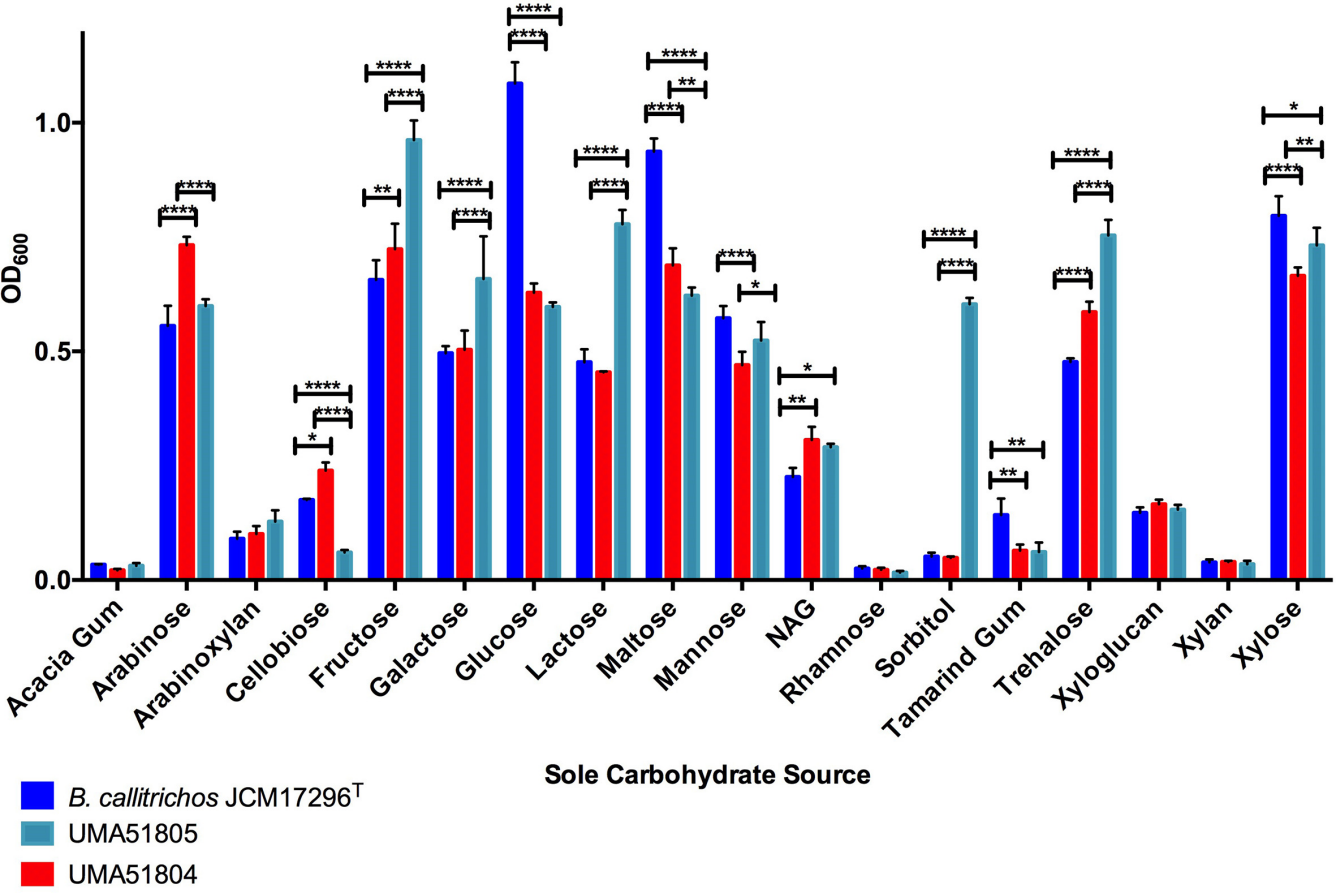
Growth of *B. callitrichos* JCM 17296^T^, UMA51804, and UMA51805 on various sole carbohydrate sources. Shown are growth profiles on acacia gum, arabinose, cellobiose, fructose, galactose, glucose, lactose, maltose, mannose, *N*-acetylglucosamine (NAG), rhamnose, sorbitol, tamarind gum, trehalose, cranberry xyloglucan, xylan, and xylose as a sole carbohydrate source. Bars represent the average final OD_600_ of biological triplicates, and error bars show the standard deviation. Sole carbohydrate sources and OD_600_ values are shown on the *x*- and *y*-axis, respectively. Significant differences among the growth profiles of strains on each carbohydrate source are computed using two-way ANOVA with significance at *P<0.05, **P<0.01, ***P<0.001, ****P<0.0001.

**Fig. 6.**
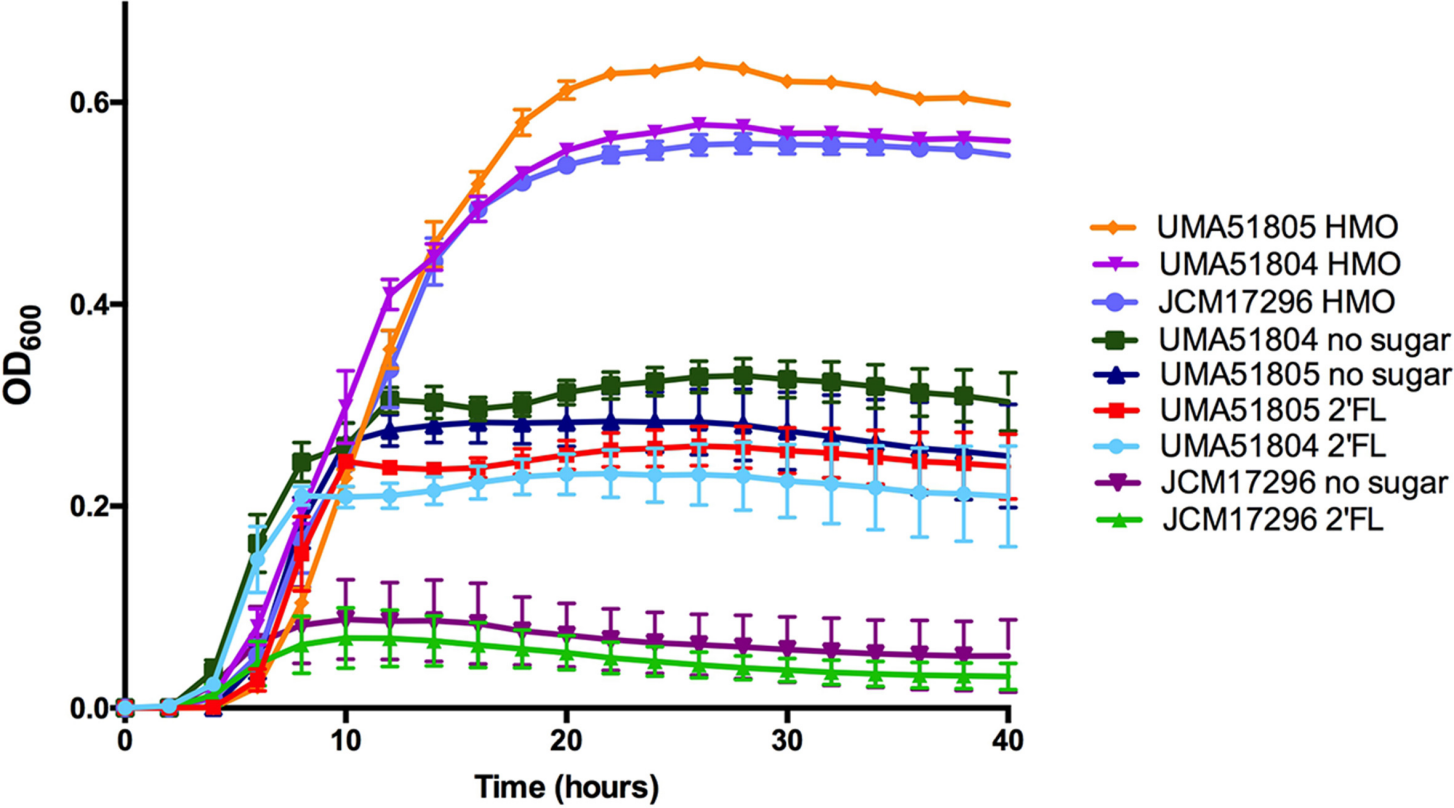
Growth of *B. callitrichos* JCM 17296^T^, UMA51804, and UMA51805 on 2′-fucosyllactose and pooled HMOs. Shown are growth profiles on 2-fucosyllactose and pooled HMOs as sole carbohydrate sources. Curves represent the average OD_600_ of biological triplicates with error bars showing the standard deviation. Growth times and OD_600_ values are shown on the *x*- and *y*-axis, respectively.

To match genomic predictions with phenotype, the three *B. callitrichos* strains (UMA51804, UMA51805, and JCM 17296^T^) were subjected to growth on carbohydrates as the sole carbohydrate source. The results of these analyses are exhibited in Fig. 5 as OD_600_ values at stationary phase. The carbohydrates to test were selected based on their relevance to bifidobacterial metabolism, the marmoset diet, and the carbohydrate utilization genes predicted within the *B. callitrichos* genome. All three strains grew on glucose, arabinose, galactose, mannose, xylose, trehalose, lactose and fructose. This is consistent with the findings of genes that are predicted to encode activities that feed these carbohydrates into central metabolism. However, there was significant variation among the three strains depending on the carbohydrate source. Accordingly, *B. callitrichos* JCM 17296^T^ grew significantly more on glucose (OD_600avg_=1.086; *P*<0.0001) than both UMA51804 (OD_600avg_=0.628) and UMA51805 (OD_600avg_=0.597). The same trend was observed with growth on maltose and tamarind gum. Conversely, UMA51804 metabolized arabinose to achieve a higher OD_600_ (OD_600avg_=0.732; *P*<0.0001) than UMA51805 (OD_600avg_=0.599) and JCM 17296^T^ (OD_600avg_=0.556). Galactose metabolism resulted in OD_600_ values that were significantly higher (OD_600avg_=0.659; *P*<0.0001) for UMA51805 than for UMA51804 (OD_600avg_=0.504) and JCM 17296^T^ (OD_600avg_=0.496). UMA51805 achieved greater cell biomass (*P*<0.0001) while subsisting on trehalose (OD_600avg_=0.754), lactose (OD_600avg_=0.778), and fructose (OD_600avg_=0.962) than UMA51804 or JCM 17296^T^. Interestingly, UMA51804 grew to a lesser extent than UMA51805 and *B. callitrichos* JCM 17296^T^ on mannose (OD_600avg_=0.470; *P*<0.001) and xylose (OD_600avg_=0.665; *P*<0.0001). UMA51804 grew more efficiently than *B. callitrichos* JCM 17296^T^ on cellobiose (UMA51804 OD_600avg_=0.239; JCM 17296^T^ OD_600avg_=0.175; *P*≤0.05), but both achieved higher final OD_600_ values than UMA51805 (OD_600avg_=0.060; *P*<0.0001). All three strains did not grow appreciably on rhamnose (total OD_600avg_=0.0214), purified acacia gum (total OD_600avg_=0.0289), and xylan (total OD_600avg_=0.0378) as the sole carbohydrate source. UMA51805 grew significantly better on sorbitol (OD_600avg_=0.603; *P*<0.0001) than UMA51804 and JCM 17296^T^. JCM 17296^T^, UMA51804 and UMA51805 all grew to an similar extent on arabinoxylan (total OD_600avg_=0.1064) and cranberry xyloglucans (total OD_600avg_=0.1557). Finally, and significantly, all three strains grew on pooled HMOs suggesting that they may metabolize similar carbohydrates in marmoset milk (Fig. 6). Interestingly, none of the three *B. callitrichos* strains grew on the typical HMO species 2′-fucosyllactose. It is not currently known to what extent 2′-fucosyllactose appears in marmoset milk. One study found that the molar concentration of fucosylated oligosaccharides in marmoset milk is under 10 % [99]. Somewhat surprising is that the two recent isolates grew moderately under negative control conditions (i.e. no carbohydrate). The assay is validated by the type strain not growing under these conditions, as well as other bifidobacterial strains (data not shown). This is intriguing as it suggests an alternative energy-generating pathway that is potentially independent of carbohydrate fermentation.

## Discussion

Bifidobacteria utilize a broad range of host-indigestible dietary carbohydrates that promote reciprocally beneficial host–microbial interactions within the gut. These traits are postulated to be a product of host–microbial coevolution, and the continued study of bifidobacteria in non-human primate species will further explicate this coevolutionary relationship. Mammalian milk maintains host-indigestible oligosaccharide utilization in infant-associated bifidobacteria, and potentially in non-human primates. A detailed analysis of primate milk oligosaccharides identified 100 oligosaccharide structures in marmoset milk, which was most similar to that of chimpanzees and humans [99]. Power *et al*. determined that the total gross energy content of marmoset milk is similar to that of several other primates, but that individual marmosets with a lower total gross energy had a higher proportion of energy from sugars [100]. These studies suggest that carbohydrates play an important role in marmoset milk. Lactating marmoset mothers may be under increased energetic stress because they often give birth to twins and immediate postpartum fertility often leads to two births per year [101, 102]. Common marmoset nursing peaks at 2 weeks and solid foods are introduced beginning at 5 weeks. Following weaning, bifidobacteria may continue to benefit their host through metabolism of other dietary carbohydrates in the adult marmoset diet. The exudatory nutritive strategy in marmosets may provide an example of evolutionary pressure to maintain gut microbial fermentation, although more research is required to examine this concept further.

Maintaining an exuditory diet presents somewhat of a nutritional challenge. Plant gums are high in carbohydrates but lower in protein. A recent analysis of marmoset feeding habits determined the carbohydrate and protein content of gums from *Anadenanthera peregrina*, a food source for marmosets in Brazil, to be 38.2 and 19.0 %, respectively [103]. This may drive marmosets to supplement their protein intake with insects [33]. The relatively low nutritional content of gum exudates increases the need of marmosets to perform highly efficient extraction of substrates for energy and biomass, to which the bifidobacteria and other members of the gut microbiome contribute. Despite these challenges, the common marmoset appears to be physiologically adapted for this purpose [32]. Small body sizes and low nutrient requirements enable common marmosets to subsist on a limited diet, and the relatively slow digestive transit time of gums allows for more complete microbial fermentation [28, 33]. In addition, the marmoset mouth and jaw architecture is hypothesized to have evolved for better access to gums within trees to ultimately provide the gut microbiota with substrates that are not fully digested by their host [104].

The composition of the marmoset gut microbiome and the influence of diet on its function are not thoroughly understood. Ley *et al*. included two marmoset species, *Callimico goeldii* and *Callithrix geoffroyi*, in a larger study of mammalian gut microbiomes and found correlations between these species and the microbiomes of other primates [15]. Another study included *C. goeldii* in an analysis of dietary strategies and found that this primate grouped with omnivores in an OTU network diagram [16]. Other studies of microbiota in marmoset feces are limited to a few culture-dependent approaches. Accordingly, high concentrations of Gram-negative bacteria (~60 %) have been isolated from the common marmoset large intestine, although this may not be a true representation of the community due to biases in culture-based approaches [105, 106]. Although gastrointestinal pathogens such as *Helicobacter* spp., enteropathogenic *Escherichia coli*, and *Clostridium difficile* have been identified in marmosets, studies of gut microbial symbionts are limited [107–109]. The previous isolation of *Lactobacillus casei* and *Bifidobacterium catenulatum* from marmoset feces suggests that gut bacteria with beneficial properties may also be active in maintaining marmoset gastrointestinal health [110]. Microbial diversity analysis (i.e. 16S amplicon sequencing) reveals that community diversity patterns at the genus level are consistent with previous studies of the human gut microbiome. This suggests that the marmoset may serve as a useful model to study the microbial diversity of the primate gut, providing a species-specific diversity signature which can be correlated with infant development and dietary changes early in life to investigate host–microbial interactions.

The microbiome of two other captive primate species, the red-shanked douc (*Pygathrix nemaeus*) and the mantled howler monkey (*Alouatta palliata*), had significantly lower alpha diversity compared to those living in the wild [111]. The similarity between the UMA51805 and JCM 17296^T^ genomes suggests that marmoset-hosted *B. callitrichos* may not vary considerably according to geographical location. However, that UMA51804 was isolated from the same marmoset host adds a measure of uncertainty to this hypothesis. The unexpectedly large variation in the UMA51804 genome may reflect adaptation to *B. callitrichos* in a captive host. An alternative explanation may be that multi-strain colonization of marmosets occurs frequently and reflects niche partitioning within non-human primates. Future studies will need to determine the extent of genomic and phenotypic plasticity in non-human primate commensals and their microbial community assemblages.

## Data bibliography

1. Sela, D. A., GenBank, NWTW00000000 (2017).
2. Sela, D. A., GenBank, NWTX00000000 (2017).
3. University of Parma, Genbank, GCA_000741175.1 (2014).

## Supplementary Data

649 Supplementary File 2
