# Supplementary material for: The comparative genomics of Bifidobacterium callitrichos reflects dietary carbohydrate utilization within the common marmoset gut
Source: Microb Genom. 2018 Jun 15;4(6):e000183. doi: 10.1099/mgen.0.000183 (PMC6096940; doi:10.1099/mgen.0.000183)
Supplement: Supplementary File 2 [file mgen-4-183-s001.pdf]

Table S1.

Transporter	Strain	Locus Tags
**Sugar ABC Transporter ATP-Binding Protein**	UMA51804	COO72_RS07555
		COO72_RS09390
		COO72_RS11870
	UMA51805	CPA40_RS00825
		CPA40_RS06250
	JCM17296[T]	BCAL_RS01040
		BCAL_RS01055
		BCAL_RS03850
**Sugar ABC Transporter Permease**	UMA51804	COO72_RS08675
		COO72_RS09380
		COO72_RS09385
		COO72_RS09870
		COO72_RS10285
		COO72_RS10290
		COO72_RS10330
		COO72_RS12020
		COO72_RS00015
		COO72_RS06525
		COO72_RS08545
		COO72_RS08675
	UMA51805	CPA40_RS10245
		CPA40_RS10280
		CPA40_RS10380
		CPA40_RS10510
		CPA40_RS10835
		CPA40_RS10840
		CPA40_RS10950
		CPA40_RS10955
		CPA40_RS00815
		CPA40_RS00820

		CPA40_RS03125
		CPA40_RS04125
		CPA40_RS05910
		CPA40_RS05915
		CPA40_RS08275
		CPA40_RS10065
	JCM17296T	BCAL_RS05410
		BCAL_RS05975
		BCAL_RS06765
		BCAL_RS06800
		BCAL_RS06850
		BCAL_RS07240
		BCAL_RS07285
		BCAL_RS07290
		BCAL_RS07615
		BCAL_RS07760
		BCAL_RS08190
		BCAL_RS08210
		BCAL_RS08445
		BCAL_RS09380
		BCAL_RS10595
		BCAL_RS01060
		BCAL_RS02165
		BCAL_RS02950
		BCAL_RS03290
		BCAL_RS03630
		BCAL_RS03840
**Sugar ABC Transporter Substrate-Binding Protein**	UMA51804	COO72_RS09875
		COO72_RS10195
		COO72_RS11860
		COO72_RS02910

		COO72_RS09395
	UMA51805	CPA40_RS00205
		CPA40_RS00325
		CPA40_RS00645
		CPA40_RS00830
		CPA40_RS04130
		CPA40_RS04630
		CPA40_RS10060
	JCM17296^T	BCAL_RS05970
		BCAL_RS07610
		BCAL_RS08205
		BCAL_RS09355
		BCAL_RS10440
		BCAL_RS02130
		BCAL_RS02135
		BCAL_RS03295
		BCAL_RS03635
		BCAL_RS03855
		BCAL_RS05400

Table S2.

Transporter	Strain	Locus Tags
**Lactose/Cellobiose PTS Transporter Subunit IIA**	UMA51804	COO72_RS06580
	UMA51805	CPA40_RS08225
	JCM17296^{T}	BCAL_RS01110
**Subunit IIB**	UMA51804	COO72_RS06575
	UMA51805	CPA40_RS08230
	JCM17296^{T}	BCAL_RS01105
**Subunit IIC**	UMA51804	COO72_RS06570
	UMA51805	CPA40_RS08235
	JCM17296^{T}	BCAL_RS01100
**Trehalose PTS Transporter Subunit IIBC**	UMA51804	COO72_RS08010
	UMA51805	CPA40_RS06365
	JCM17296^{T}	BCAL_RS06200
**N-Acetylglucosamine PTS Transporter Subunit IIABC**	UMA51804	COO72_RS07250
		COO72_RS07255
	UMA51805	CPA40_RS03800
		CPA40_RS03805
	JCM17296^{T}	BCAL_RS11835
		BCAL_RS11840
**Beta-Glucoside PTS Transporter Subunit EIIBCA**	UMA51804	COO72_RS09735
		COO72_RS10100
	UMA51805	CPA40_RS03330

Table S3.

	$JCM17296^T$	UMA51804	UMA51805
**Carbohydrate-Binding Molecules**	CBM22 (1)	CBM13 (4)	CBM13 (4)
	CBM32 (1)	CBM22 (1)	CBM22 (1)
	CBM48 (5)	CBM32 (1)	CBM32 (1)
	CBM50 (3)	CBM48 (3)	CBM48 (5)
	CBM67 (2)	CBM50 (3)	CBM50 (1)
			CBM67 (2)
**Carbohydrate Esterases**	CE1 (6)	CE1 (4)	CE1 (5)
	CE10 (1)	CE10 (2)	CE10 (3)
	CE2 (1)	CE2 (1)	CE2 (1)
	CE3 (1)	CE3 (1)	CE3 (2)
	CE4 (2)	CE4 (2)	CE4 (2)
	CE6 (2)	CE6 (2)	CE6 (3)
	CE9 (2)	CE9 (2)	CE9 (3)
**Glycosyl Hydrolases**	GH1 (3)	GH1 (5)	GH1 (2)
	GH109 (2)	GH109 (3)	GH109 (4)
	GH115 (1)	GH112 (1)	GH115 (2)
	GH125 (1)	GH115 (2)	GH123 (1)
	GH127 (2)	GH123 (2)	GH125 (1)
	GH13_11 (2)	GH127 (2)	GH127 (2)
	GH13_13 (1)	GH13_11 (2)	GH13_11 (2)
	GH13_18 (1)	GH13_18 (1)	GH13_13 (1)
	GH13_29 (1)	GH13_29 (1)	GH13_18 (1)
	GH13_3 (1)	GH13_3 (1)	GH13_29 (1)
	GH13_30 (3)	GH13_30 (1)	GH13_3 (1)
	GH13_31 (1)	GH13_31 (1)	GH13_30 (2)
	GH13_4 (1)	GH13_4 (1)	GH13_31 (1)
	GH13_9 (1)	GH13_9 (1)	GH13_4 (1)

GH13 (2)	GH13 (2)	GH13_9 (1)
GH16 (1)	GH142 (1)	GH13 (2)
GH2 (4)	GH2 (4)	GH2 (4)
GH20 (1)	GH23 (1)	GH20 (1)
GH23 (2)	GH25 (2)	GH23 (2)
GH25 (2)	GH27 (2)	GH25 (1)
GH27 (4)	GH3 (2)	GH27 (2)
GH28 (2)	GH30 (2)	GH28 (2)
GH29 (1)	GH31 (2)	GH3 (5)
GH3 (3)	GH32 (1)	GH30_2 (1)
GH30 (2)	GH35 (1)	GH30 (2)
GH31 (3)	GH36 (4)	GH31 (3)
GH32 (1)	GH42 (3)	GH32 (1)
GH36 (3)	GH43_22 (4)	GH36 (2)
GH38 (3)	GH43_24 (2)	GH38 (3)
GH42 (4)	GH43 (1)	GH4 (1)
GH43_11 (1)	GH5_35 (5)	GH42 (5)
GH43_12 (1)	GH51 (4)	GH43_11 (1)
GH43_22 (3)	GH59 (1)	GH43_12 (1)
GH43_24 (1)	GH77 (2)	GH43_22 (3)
GH43_26 (1)		GH43_24 (1)
GH43_27 (1)		GH43_26 (1)
GH43_4 (2)		GH43_27 (1)
GH5_18 (1)		GH43_4 (2)
GH50 (1)		GH5_18 (1)
GH51 (2)		GH5_35 (2)
GH59 (1)		GH5_47 (1)
GH77 (2)		GH50 (1)
GH78 (2)		GH51 (2)

	GH85 (1)		GH59 (1)
			GH77 (2)
			GH78 (2)
			GH78 (1)
			GH85 (1)
**Glycosyl Transferases**	GT2 (15)	GT2 (16)	GT2 (11)
	GT28 (1)	GT28 (2)	GT27 (1)
	GT32 (1)	GT35 (1)	GT28 (1)
	GT35 (1)	GT39 (1)	GT35 (1)
	GT39 (1)	GT4 (3)	GT4 (4)
	GT4 (5)	GT5 (1)	GT5 (1)
	GT5 (1)	GT51 (2)	GT51 (2)
	GT51 (2)	GT8 (2)	GT8 (2)
	GT83 (1)	GT81 (1)	GT81 (1)
		GT83 (2)	GT83 (2)
**Polysaccharide Lyases**	PL27 (1)	PL27 (1)	PL27 (2)

Table S4.

	CAZy Category
**Shared Extracellular Carbohydrate-Active Enzymes (CAZy)**	Carbohydrate-Binding Molecule Family 22<br>Glycosyl Hydrolase Family 51<br>Carbohydrate Esterase Family 4<br>Glycosyl Hydrolase Family 25
**JCM17296^T**	Glycosyl Hydrolase 43_27<br>Glycosyl Hydrolase 43_26<br>Carbohydrate-Binding Molecule 50
**UMA51804**	Carbohydrate-Binding Molecule 13<br>Glycosyl Hydrolase 5_35<br>Glycosyl Hydrolase 59<br>Carbohydrate-Binding Molecule 50
**UMA51805**	Glycosyl Hydrolase 5_47<br>Glycosyl Hydrolase 43_26<br>Glycosyl Hydrolase 43_27<br>Carbohydrate-Binding Molecule 13<br>Glycosyl Hydrolase 5_35

Figure S1.

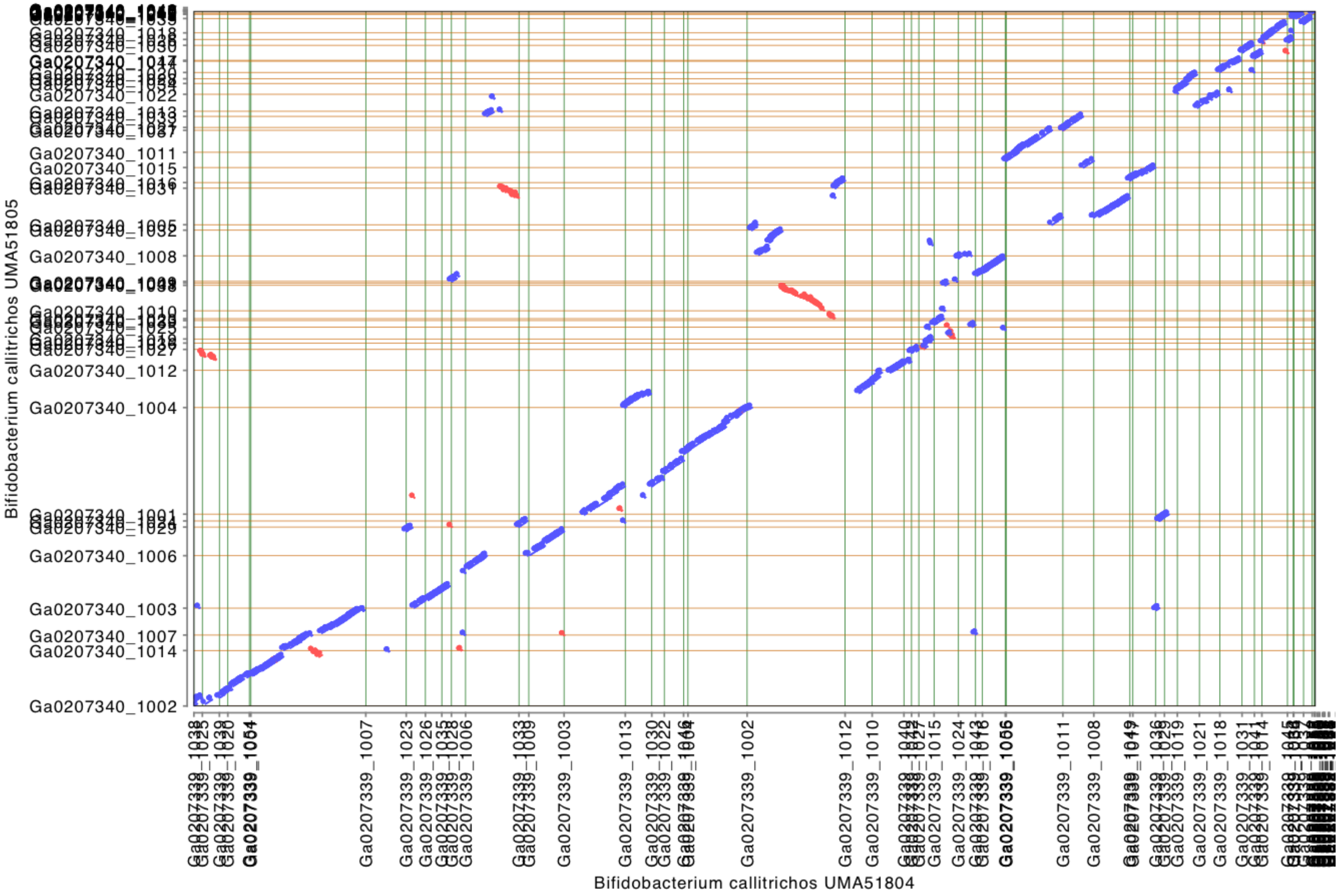
Bifidobacterium callitrichos UMA51805
Ga0207340_1018
Ga0207340_1022
Ga0207340_1011
Ga0207340_1015
Ga0207340_1008
Ga0207340_1010
Ga0207340_1012
Ga0207340_1004
Ga0207340_1006
Ga0207340_1003
Ga0207340_1007
Ga0207340_1014
Ga0207340_1002
Ga0207339_1007
Ga0207339_1023
Ga0207339_1026
Ga0207339_1006
Ga0207339_1003
Ga0207339_1013
Ga0207339_1002
Ga0207339_1012
Ga0207339_1010
Ga0207339_1015
Ga0207339_1024
Ga0207339_1011
Ga0207339_1008
Ga0207339_1019
Ga0207339_1021
Ga0207339_1018
Bifidobacterium callitrichos UMA51804

Figure S2.

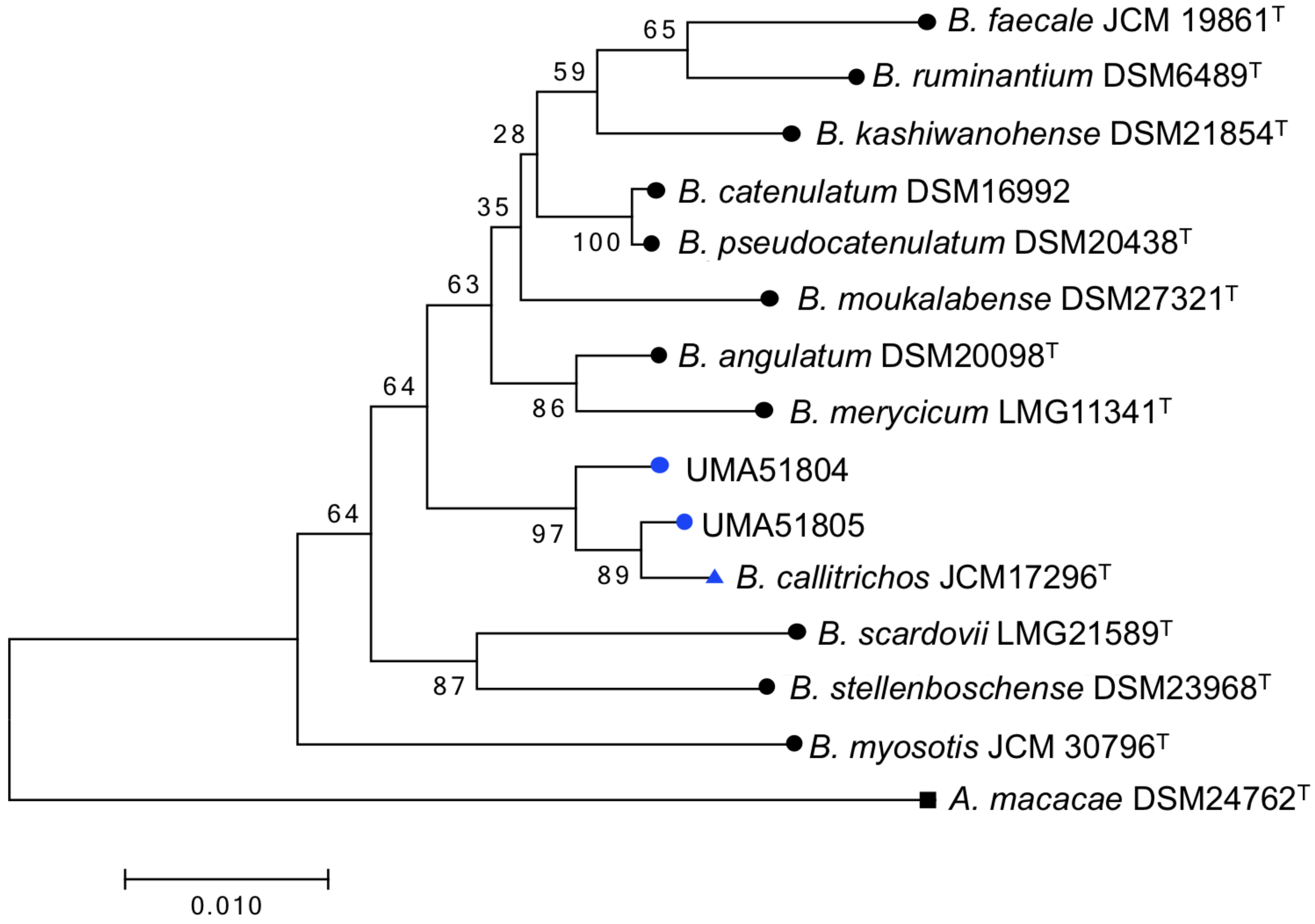
65
59
28
35
100
63
64
86
64
97
89
87
B. faecale JCM 19861T
B. ruminantium DSM6489T
B. kashiwanohense DSM21854T
B. catenulatum DSM16992
B. pseudocatenulatum DSM20438T
B. moukalabense DSM27321T
B. angulatum DSM20098T
B. merycicum LMG11341T
UMA51804
UMA51805
B. callitrichos JCM17296T
B. scardovii LMG21589T
B. stellenboschense DSM23968T
B. myosotis JCM 30796T
A. macacae DSM24762T
0.010

Figure S3.

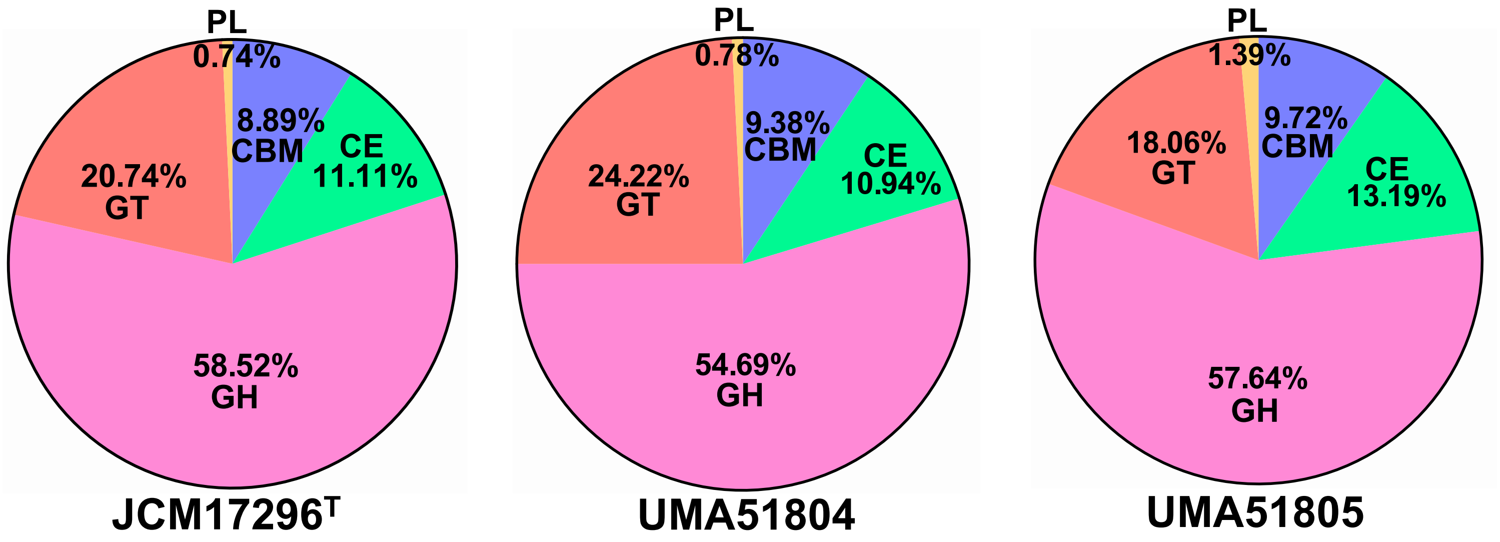
PL
0.74%
8.89%
CBM
CE
11.11%
20.74%
GT
58.52%
GH
JCM17296T
PL
0.78%
9.38%
CBM
CE
10.94%
24.22%
GT
54.69%
GH
UMA51804
PL
1.39%
9.72%
CBM
CE
13.19%
18.06%
GT
57.64%
GH
UMA51805

Figure S4.

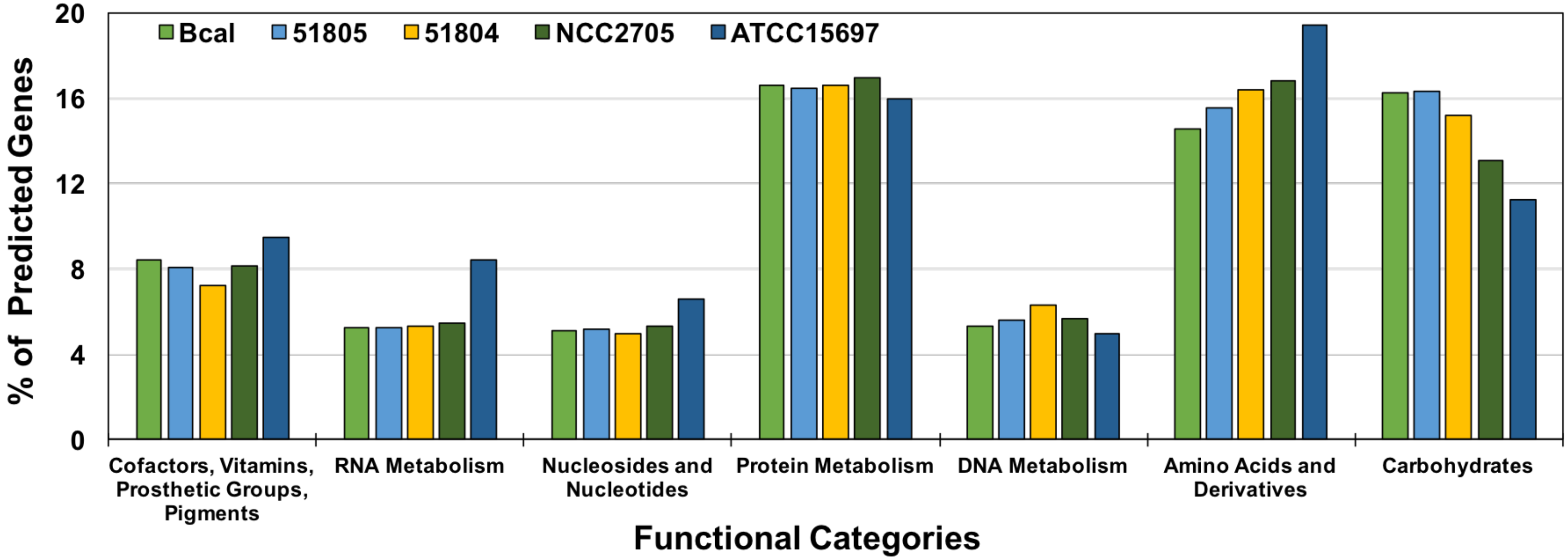
20
16
12
8
4
0
% of Predicted Genes
Bcal
51805
51804
NCC2705
ATCC15697
Cofactors, Vitamins, Prosthetic Groups, Pigments
RNA Metabolism
Nucleosides and Nucleotides
Protein Metabolism
DNA Metabolism
Amino Acids and Derivatives
Carbohydrates
Functional Categories

Supplementary Table and Figure Legends.

Supplementary Table 1. Multiple sugar ABC transporters shared by the type strain *B. callitrichos* JCM17296^{T}, UMA51804, and UMA51805 isolates identified in this study.

Supplementary Table 2. PTS transporter systems shared by the type strain *B. callitrichos* JCM17296^{T}, UMA51804, and UMA51805 isolates identified in this study.

Supplementary Table 3. Carbohydrate-active enzyme families in the type strain *B. callitrichos* JCM17296^{T}, UMA51804, and UMA51805 isolates identified in this study. The numbers in parentheses next to each enzyme family corresponds to the number of genes matching with each family. Abbreviations are as follows: GH, glycosyl hydrolase, CE, carbohydrate esterase, CBM, carbohydrate-binding molecule, GT, glycosyl transferase, PL, polysaccharide lyase.

Supplementary Table 4. Carbohydrate-active enzyme families with predicted extracellular localization in the type strain *B. callitrichos* JCM17296^{T}, UMA51804, and UMA51805 isolates identified in this study.

Fig. S1. Dot-plot analysis of genome comparison to identify regions of close similarities among the isolates UMA51804 and UMA51805. Dot Plot employs Mummer to generate dotplot diagrams between the two genomes. It uses the six frame amino acid translation of the DNA input sequences (PROmer) for comparing genomes with dissimilar sequences. The dotplot graph shows blue points for the regions of similarity found on parallel strands (fplot) and red points for regions of similarity found on antiparallel strands (rplot). The dot for each point shows the coordinates and scaffolds of the alignment. Chromosomal neighborhood of the alignment of the two genomes can be downloaded from the online versions of the dotplot by clicking on the points.

Fig. S2. Neighbor-joining phylogenetic tree of members of the genus *Bifidobacterium* originating from non-human primates and isolates UMA51804 and UMA51805. Phylogenetic analysis based on 16S rRNA gene sequences for type strains and isolates UMA51804 and UMA51805. Bootstrap confidence values obtained with 100 resamplings are given at the branch point. Microbial isolated identified in this study are shown as blue circles closely clustered with type strain B. callitrichos JCM 17296T (blue triangle). All other non-human primates Type strains are represented with black circles. *Alloscardovia macacae* (DSM24762) was used as the outgroup species within Bifidobacterium family (black square). A total of 13 Type strains and 2 microbial isolates identified in this study are included in this analysis. The 16S rRNA gene phylogenetic tree is generated using MEGA.

Figure S3. Pie charts representation showing the proportion of each carbohydrate-active enzyme category found in the type strain *B. callitrichos* JCM17296^T, UMA51804, and UMA51805 isolates identified in this study. Abbreviations are as follows: GH, glycosyl hydrolase, CE, carbohydrate esterase, CBM, carbohydrate-binding molecule, GT, glycosyl transferase, PL, polysaccharide lyase.

Figure S4. Bar chart representation for the predicted functional categories of genes in *B. callitrichos* JCM17296^T, UMA51805, UMA51804, *B. longum* subsp. *longum* NCC2705, and *B. longum* subsp. *infantis* ATCC15697. The percentage of protein-coding genes in each genome are shown for the seven most abundant functional categories. The predicted genes are identified in each genome based on the RAST annotations using the SEED database.
